# Performance of Machine Learning Classifiers in Classifying Stunting among Under-Five Children in Zambia

**DOI:** 10.3390/children9071082

**Published:** 2022-07-20

**Authors:** Obvious Nchimunya Chilyabanyama, Roma Chilengi, Michelo Simuyandi, Caroline C. Chisenga, Masuzyo Chirwa, Kalongo Hamusonde, Rakesh Kumar Saroj, Najeeha Talat Iqbal, Innocent Ngaruye, Samuel Bosomprah

**Affiliations:** 1 African Centre of Excellence in Data Science, College of Business Studies Kigali, University of Rwanda, Gikondo—Street, KK 737, Kigali P.O. Box 4285, Rwanda; 2 Enteric Disease and Vaccines Research Unit, Centre for Infectious Disease Research in Zambia, Lusaka P.O. Box 34681, Zambia; roma.chilengi@cidrz.org (R.C.); michelo.simuyandi@cidrz.org (M.S.); caroline.chisenga@cidrz.org (C.C.C.); masuzyo.chirwa@cidrz.org (M.C.); kalongo.hamunsonde@cidrz.org (K.H.); samuel.bosomprah@cidrz.org (S.B.); 3 Department of Community Medicine, Sikkim Manipal Institute of Medical Sciences (SIMMS) Sikkim Manipal University, Gangtok 03592, India; rakesh.saroj@bhu.ac.in; 4 Department of Paediatrics and Child Health, Biological and Biomedical Sciences, Aga Khan University Hospital, Karachi 74800, Pakistan; najeeha.iqbal@aku.edu; 5 College of Science of Technology, University of Rwanda, KN 7 Ave, Kigali P.O. Box 4285, Rwanda; igaruye@gmail.com; 6 Department of Biostatistics, School of Public Health, University of Ghana, Accra P.O. Box LG13, Ghana

**Keywords:** stunting, machine learning, random forest, Naïve Bayesian, ZDHS

## Abstract

Stunting is a global public health issue. We sought to train and evaluate machine learning (ML) classification algorithms on the Zambia Demographic Health Survey (ZDHS) dataset to predict stunting among children under the age of five in Zambia. We applied Logistic regression (LR), Random Forest (RF), SV classification (SVC), XG Boost (XgB) and Naïve Bayes (NB) algorithms to predict the probability of stunting among children under five years of age, on the 2018 ZDHS dataset. We calibrated predicted probabilities and plotted the calibration curves to compare model performance. We computed accuracy, recall, precision and F1 for each machine learning algorithm. About 2327 (34.2%) children were stunted. Thirteen of fifty-eight features were selected for inclusion in the model using random forest. Calibrating the predicted probabilities improved the performance of machine learning algorithms when evaluated using calibration curves. RF was the most accurate algorithm, with an accuracy score of 79% in the testing and 61.6% in the training data while Naïve Bayesian was the worst performing algorithm for predicting stunting among children under five in Zambia using the 2018 ZDHS dataset. ML models aids quick diagnosis of stunting and the timely development of interventions aimed at preventing stunting.

## 1. Introduction

Stunting is still one of the most serious health and welfare issues globally. In 2019, about 21.3% of children under five years of age were estimated to be stunted globally, and two out of five stunted children live in Africa [1]. Between 2000 and 2020, the global prevalence of stunting fell from about 30.3% to 22% [2]. Despite the fall in the magnitude of stunting, the prevalence of stunting has remained high in the sub-Saharan region [3]. In Zambia, the prevalence of stunting among children under five was estimated to be 35% in 2019 [4]. Prevalence of stunting is classified as very low, low, medium, high and very high if it is <2.5%, between 2.5 and 10%, between 10 and 20%, between 20 and 30% and above 30%, respectively [5].

Stunting among children is associated with short-term and long-term health and social outcomes. Mortality and morbidity are among the most common short-term effects of stunting [6,7]. Some long-term effects of childhood stunting include poor cognitive development, poor school performance, delay in motor development and poor maternal health outcomes [8,9,10].

Over the years, classical statistical models have been used to identify factors that are independently associated with stunting among children under five [11,12,13]. However, these methods tend not to be robust in situations where the number of covariates is more than observations and when there is multi-correlation among variables. Furthermore, they follow strict assumptions about the data and the data generating process, such as the distribution of errors and additivity of parameters with linear predictors, which may not hold in real-life [14]. Compared to classical models, machine learning models overcome the analytical challenges of a large number of covariates and multicollinearity, require fewer assumptions, incorporate high dimensional data and thus produce a more flexible relationship between predictor and outcome variables [15]. These methods have been applied in predicting malnutrition using different datasets [16,17,18,19]. Furthermore, machine learning methods have been shown to be superior to classical statistical methods when solving classification problems [20].

In this study, we aimed to train, evaluate and select the best machine learning classifier for predicting stunting among children under five years in Zambia and identifying important variables in the prediction of stunting using the 2018 Zambia Health Demographic survey dataset. This model would serve as the basis for developing an intelligent model for diagnosing or predicting stunting, and features identified as important predictors of stunting would serve as variables to target when designing interventions aimed at preventing stunting among children under five in Zambia.

## 2. Materials and Methods

### 2.1. Data Source and Research Workflow

We utilized nutrition data from the 2018 Zambian demographic health survey (ZDHS) conducted by the Zambian Statistical Agency in collaboration with USAID. The survey was conducted such that it is representative of the Zambian population. It employed a stratified two-stage sampling design. The strata were defined by province and residence (i.e., rural-urban)—there are 10 provinces in Zambia, giving a total of 20 strata. The first stage involved selecting clusters defined as Enumeration Areas (EAs). For each stratum, EAs were selected using a probability proportional to size algorithm. In the second stage, a fixed number of households were selected from each EA using a systematic sampling technique. Details of the sampling methods are described in [4].

The ZDHS was performed per the Declaration of Helsinki and approved by an appropriate ethics committee. Ethical clearance was obtained from the Ethical Review Committee of the Ministry of Health, the University of Zambia Biomedical Ethics Committee and the Tropical Disease Research Centre Ethics Committee. The 2018 ZDHS survey was approved on the 27th of March 2018 by the TDRC ethics committee under protocol number STC/2018/6 and by the IRB on 6th March 2018 under protocol number 132989.0.000.ZM.DHS.02. Informed consent was obtained from participants before data collection. Permission was sort and granted on 22 March 2021 from the DHS program to use this dataset for research, and the dataset was accessed through IPUMS [21]. All data were anonymized before the authors received the data. All methods were performed following the relevant guidelines and regulations.

**Figure 1:**
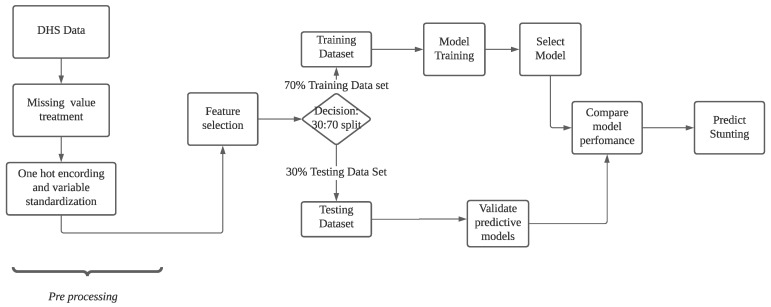
Workflow chart.

Figure 1 below depicts the research workflow. The pre-processing was followed by the feature selection, which led to a 30:70 split in the decision. In the 70% of the dataset (training dataset), model training was conducted, and then a model was selected and performance evaluated, while in the 30% of the data (testing dataset), the predictive models were validated and model performance compared to predict stunting. 

### 2.2. Pre-Processing

In this study, the target feature was stunting, which was defined based on the WHO standard, of height-for-age Z-score (HAZ) < −2 standard deviations (SD) [5]. The mothers’ socioeconomic, demographic characteristics, and feeding practices were selected as features from the ZDHS database. Missing instances were dropped from the analysis. Further, continuous variables were standardized to a standard normal distribution. Then we applied ordinal and one-hot encoding to ordinal and non-ordinal categorical features, respectively.

### 2.3. Feature Selection

**Figure 2:**
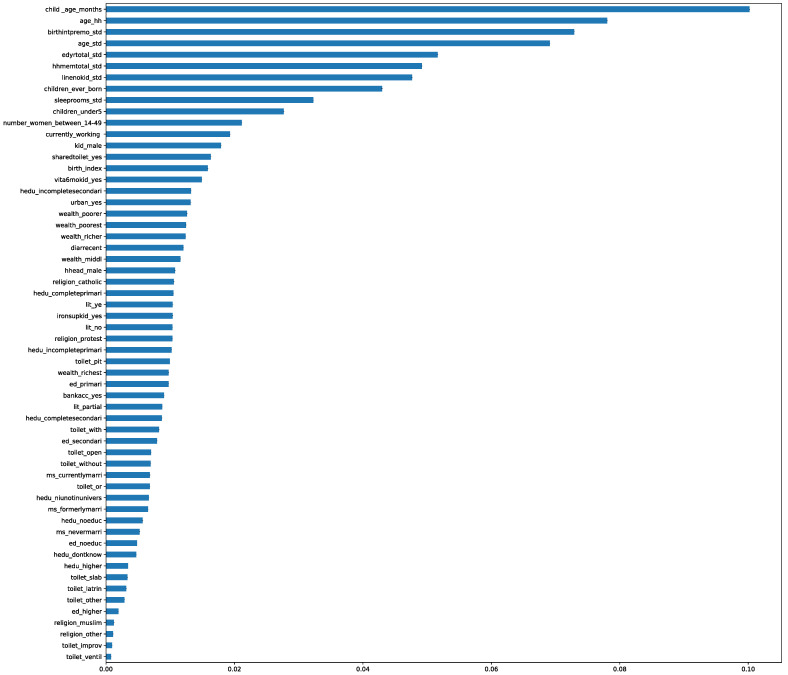
Feature importance score.

Random Forest (RF) feature selection was used to select important features. Tulukdar (2020) recommends the use of RF feature selection when building a predictive model for malnutrition [19]. The model assigns an importance score to each feature, and features that had an importance score less than the average importance score were not included in the model. Figure 2 below shows each feature and its associated importance score.

### 2.4. Model Training

We split the data into 70% training and 30% testing dataset. We evaluated five widely used machine learning classifiers, namely: Logistic regression (LR), Random Forest (RF), Naïve Bayesian (NB), Support Vector Machine (SVM) and eXtreme Gradient Boosting (Xg boost), implemented in scikit learn [22], to predict the probability of stunting.

#### 2.4.1. Logistic Regression

Logistic regression is a supervised machine learning algorithm used to solve classification problems [23]. It is a parametric method that assumes a Bernoulli distribution of the target variable and the independence of the observations [24]. Logistic regression is a common regression model used to predict class membership probabilities and is defined as:

$$\log it(Y_{i}=1|X^{T})=\log\frac{\pi_{i}}{1-\pi_{i}}=\beta_{0}+\beta X^{T}$$

where $\pi_{i}=pr(Y_{i}=1|X_{i}^{T})$ is the conditional probability of an observation being in class 1 given the covariates *X*, $\beta_{0}\;$is the intercept, and β is the vector of regression coefficients. The logistic regression model can be fitted using the maximum likelihood method.

#### 2.4.2. Random Forest

Random forest (RF) is an ensemble method consisting of a collection of tree-based structured classifiers [17]. RF is used for classification, regression and dimension reduction. It is efficient even in instances where there are more variables than observations. To classify, RF builds many decision trees; each tree makes its independent classification. The RF chooses the class that has the most votes.

#### 2.4.3. Naïve Bayesian (NB)

Naïve Bayesian is a collection of machine learning classification algorithms built on the Bayes theorem. These algorithms are built on two main assumptions; the first is that every pair of features being classified is independent of the other, and the second is that each makes an independent and equal contribution to the outcome. Though simple, the NB has high functionality [25,26]. For a binary outcome, a Bernoulli Naïve Bayesian algorithm is appropriate. NB formula is given as:

$$P\left(y|X\right)=\frac{P\left(X|y\right)P\left(y\right)}{P\left(X\right)}\;$$

where *X* is the independent predictors and *P(X)* is the predictors’ prior probability, also referred to as evidence. P(y|X) is the probability of label y given predictors *X*. This is also referred to as the posterior probability, and *P(y)* is referred to as the probability before evidence is seen or the prior. P(X|y) is known as the likelihood.

#### 2.4.4. Support Vector Machine

A support vector machine is a supervised machine learning algorithm whose goal is identifying a reproducible hyperplane of n-dimensions that maximizes the distance between support vectors of two class labels. SVM models are effective when there are more variables than samples and still effective when the sample size is small. Although it is memory efficient, SVM models do not provide probability estimates directly but through an expensive five-fold cross-validation process [27,28]

#### 2.4.5. XG Boost

XG boost, also known as eXtreme Gradient Boosting, is a decision tree-based ensemble machine learning algorithm that uses a gradient boosting framework (Friedman et al. (2000)) [29]. Boosting involves combining weak classifiers to produce a powerful averaged classifier, and it is also a variance reduction technique. It can be applied to both classification and prediction problems. The boosted decision trees are designed for optimal speed and improved model performance [30,31].

### 2.5. Model Performance Evaluation

We plotted the reliability graphs to evaluate the performance of each model. We later calibrated the predicted probabilities to reflect the occurrence of stunting in the data using the isotopic regression. The major strength of isotopic regression as a calibration method is that it can correct any monotonic distortion [32]. We determined the optimal probability threshold, on the calibrated probabilities, for classifying an instance as stunted or not.

We used 3-fold cross-validation on the training set, and the performance was estimated on the testing set. Models were evaluated based on the *F*1 score, Cohen’s kappa, the area under the precision–recall curve (AUC-PR) and the sensitivity and specificity of each model. Data analysis was conducted using Python version 3.10.2 [33]. Data were summarized using proportion and a chi-squared test of independence to test for any association between stunting. Statistical analysis was set at a *p*-value < 0.05.

*F*1 score is the harmonic mean of the precision and recall of the model is calculated using the formula below:

$$F1=\frac{2}{\frac{1}{recall}\;X\;\frac{1}{precision}}=2\;X\;\frac{precison\;X\;recall}{precision+recall}=\frac{tp}{tp+\frac{1}{2}\;\left(fp+fn\right)}\;$$

where *recall*, also known as sensitivity, is the proportion classified as positive among all the positive instants in the dataset. *Precision* is the proportion of true positive instances among the instances that the model has predicted as positive. True positive (*tp*) is the number of positive instances that are classified as positive by the model. False-positive (*fp*) is the number of negative instances that are classified as negative by the model.

Cohen’s kappa score is a metric used to measure inter-rater agreement. Cohen kappa takes into account agreement that may exist between two measures due to chance, and this is one of the reasons that makes it a robust measure for evaluating classification models.

## 3. Results

### 3.1. Characteristics of Participants

In our dataset, there were a total of 6799 children under five years. Of these, 3421 (50.3%) were male, and 5253 (77.3 %) were aged 12 months and above (Table 1). A child’s age, wealth index, region and gender were associated with stunting. About 37.6% of children aged between 12 months and 59 months were stunted compared to 22.6% of the aged less than 12 months. A total of 38.2% of children born to a mother without any formal education were stunted. Most of the children were from poor families (48%), and 38.3% of these were stunted. The prevalence of stunting was 34.2%.

### 3.2. Features Selected Using Random Forest Feature Selection

Figure 2 shows the importance score for each feature in the dataset. The child’s age in months had the highest importance score, while having an improved toilet facility had the least importance score. Only 13 out of 58 features were included. Child’s gender, mothers age, age of household head, child’s age (months), number of sleeping rooms in the household, number of women between 15 and 49 in the household, the birth interval in months, shared toilet, mother’s current employment status, years of education, number of children under five in the household and the total number of children ever born form a mother, were features that were selected as predictors of stunting among children under five.

Figure 2 shows the feature importance score for each feature included in the analysis dataset in descending order.

### 3.3. Comparison of Efficiency of Machine Learning Algorithm

**Figure 3:**
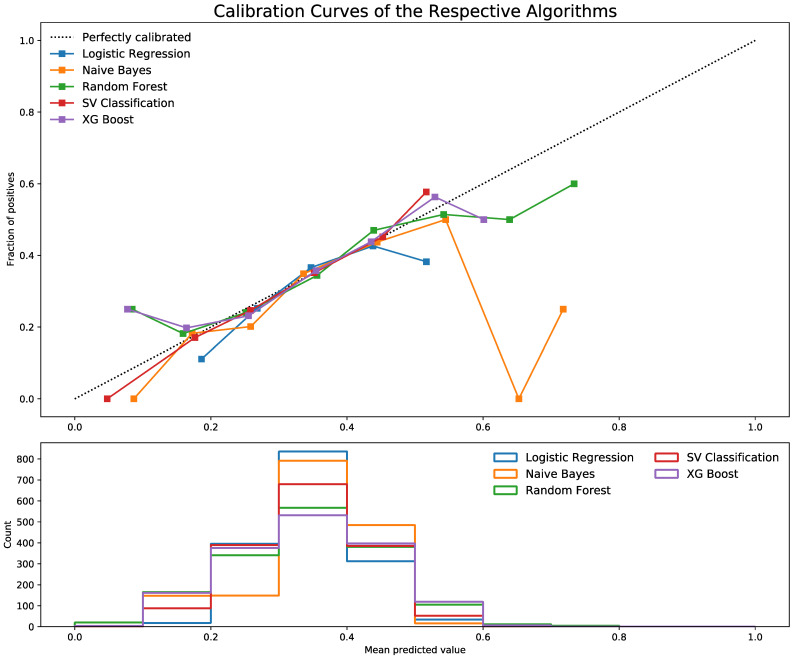
Calibration curve for ML algorithms.

Figure 3 shows the calibration curves for each machine learning algorithm with the average predicted probability for each bin on the *x*-axis and the fraction of positive classes in each bin on the *y*-axis, and below the count of positives in each bin.

All five models showed divergence from the perfectly calibrated line (Figure 2). This implies the need to calibrate the predicted probability distribution. SV Classification was the worst-performing in all bins compared to other models. After calibrating the predicted probabilities, all models improved and were better aligned with the perfect calibration curve, except for the Naïve Bayes model. The model was dropped because it was inaccurate even after calibration (Figure 3).

We presented the predictive performance of each classifier on the training and test dataset (Table 2). Logistic regression was the least accurate model both on the training and the test dataset, with an accuracy of 44.7% and 45.9%, respectively, whereas Random Forest Model was superior, with a training and testing accuracy of 79.2% and 61.6%, respectively. Random Forest Model had the large *F*1 score in training and the least in the testing dataset, while Logistic regression had the least *F*1 score in the training dataset; it had the highest score in the testing data. Logistic regression had a Cohen’s kappa score of 0.07 and 0.08 in the training and testing dataset, respectively. Random forest had a Cohen’s kappa score of 0.55, the highest in the training dataset, and 0.178, the second largest in the testing dataset (Table 2).

We showed the average precision and recall for each machine learning classifier (Table 3). XB boost had precision and recall of 60%, and logistic regression had a precision of 58% and a recall of 55%. Random forest and CV classification had 58% and 59% precision and recall.

## 4. Discussion

In our study, we identified the random forest model as the model with the highest predictive accuracy for stunting among children under five years in Zambia using the ZDHS 2018 data. Despite Random Forest and XG Boost performing better than the traditional logistic regression, logistic regression still retains interpretability as the main advantage it has over the other ML algorithm. Similar studies used ML algorithms to predict the nutritional status of children using demographic health survey data [18,19,20,32]. Our results are similar to the findings of [19], which implicate the RF algorithm to be a superior predictor of stunting.

Further, we identified features that are important in predicting stunting among children under five in Zambia. Some of the identified features, such as Mother’s education, mother’s age, age of the child, family size and residence, were identified commonly as predictors of stunting [11,16,34,35]. We suggest the need to collect more features for predicting stunting as opposed to keeping only those variables collected in the ZDHS. Though powerful, the ML models have a limitation in that they do not come with odds ratios or coefficients to indicate the direction of the relationship of the important features. Knowing the direction of the association of each importance would enhance the design and implementation of interventions aimed at preventing stunting among children under 5 years.

Despite being a widely used measure for stunting, HAZ (Z-score < −2) is an arbitrary cut-off for stunting, which may have little clinical significance [36]. Our study presents an opportunity to look at individualized risk assessment for stunting among children. Further, our study took into consideration key social, economic and environmental factors that may be key determinants of a child’s nutrition status compared to HAZ, which only takes into consideration the age, height and weight of children, which is one major strength of the ML algorithm in predicting health outcomes [37].

The major strength of this study is using the ZDHS dataset; the ZDHS applies a sampling method that is robust and, as such, is representative of the under-five population of children from both rural and urban parts of Zambia. Despite applying an optimized machine classification model, the study only applied five mostly used algorithms, yet more algorithms such as the ones used in [38]. Machine learning algorithms use features based on their importance or contribution to the model and not necessarily causal effect.

We recommend further research or developing a prognostic model for stunting using longitudinal data. This would help in the development of timely interventions aimed at preventing stunting. Although the ZDHS dataset is very representative of the Zambian population of children under five, it may not contain features that would be very instrumental in building a predictive model for stunting; this is because data are mainly not collected for the sole purpose of a study of this nature. ML learning aids timely diagnosis of stunting and timely design, evaluation and deployment of mitigation measures. Since the effects of stunting are unreversible in the long run, having a machine learning-based risk score would aid the treatment of malnutrition.

## 5. Conclusions

The results suggest that the Random Forest machine learning algorithm has the highest predictive accuracy for stunting compared to other models applied in this study. We also identified the children’s and their mothers’ social and economic features that are important predictors of stunting among children under five.

## Figures and Tables

**Table 1:** Percent of stunted children by background characteristics.

Characteristics	Number of Children (% of Total)	Stunted, *n* (%)	*p*-Value
Child’s age			
12 months	1546 (22.7)	350 (22.6)	<0.001
>12 months	5253 (77.3)	1977 (37.6)	
Gender			
Male	3421 (50.3)	1075 (31.4)	<0.001
Female	3378 (49.7)	1252 (37.1)	
Mother’s Age (years)			
15–24	1938 (28.5)	697 (36)	0.088
25–34	3181 (46.8)	1084 (34.1)	
35–49	1680 (24.7)	546 (32.5)	
Region			
Urban	4875 (71.7)	1734 (35.6)	<0.001
Rural	1924 (28.3)	593 (30.8)	
Mother’s Education			
No formal education	738 (10.9)	282 (38.2)	<0.001
Primary	3722 (54.7)	1390 (37.3)	
Secondary	2045 (30.1)	615 (30.1)	
Higher	294 (4.3)	40 (13.6)	
Mothers Current work			
No	3505 (51.6)	1172 (33.4)	0.158
Yes	3294 (48.4)	1155 (35.1)	
Wealth Index			
Poor	3261 (48)	1250 (38.3)	<0.001
Middle	1308 (19.2)	453 (34.6)	
Richer	2230 (32.8)	624 (28)	
Religion			
Muslim	39 (0.6)	17 (43.6)	0.183
Catholic	1093 (16.1)	399 (36.5)	
Protestant	5603 (82.4)	1891 (33.7)	
Other	64 (0.9)	20 (31.3)	
Toilet type			
Unhygienic	4265 (62.7)	1428 (33.5)	0.094
Hygienic	2534 (37.3)	899 (35.5)	
Total	6799 (100)	2327 (34.2)	

**Table 2:** Accuracy score for each classification model in predicting stunting.

Model	Train F1	Test F1	Train Cohen’s Kappa	Test Cohen’s Kappa	Train PR-AUC	Test PR-AUC	Train Accuracy	Test Accuracy
Logistic Regression	0.5298	0.5411	0.0797	0.0833	0.3728	0.3858	0.4471	0.4592
Random Forest	0.717	0.4826	0.5535	0.178	0.5992	0.4134	0.7921	0.6162
SV Classification	0.6083	0.523	0.3106	0.1486	0.4611	0.4051	0.6402	0.5583
XG Boost	0.6006	0.5381	0.3019	0.188	0.4566	0.4192	0.6385	0.5851

**Table 3:** Precision and recall for each machine learning algorithm.

Model	Precision Negative	Precision Positive	Average Precision	Recall Negative	Recall Positive	Average Recall
Logistic Regression	0.78	0.39	0.585	0.22	0.89	0.555
Random Forest	0.71	0.47	0.59	0.68	0.5	0.59
SV Classification	0.73	0.43	0.58	0.49	0.67	0.58
XG Boost	0.75	0.45	0.6	0.54	0.67	0.605

## Data Availability

The data used for this study can be accessed with permission from IPUMS DHS https://www.idhsdata.org/idhs/ (accessed on 22 March 2021).

## References

[1] World Health Organization (2021). Levels and Trends in Child Malnutrition: Geneva, 2021.

[2] World Health Organization (2020). Levels and Trends in Child Malnutrition: Geneva, 2020.

[3] Quamme S.H., Iversen P.O. (2022). Prevalence of child stunting in Sub-Saharan Africa and its risk factors. Clin. Nutr. Open Sci..

[4] Zambia Statistics Agency, Ministry of Health (MOH) [Zambia] (2019). Zambia Demographic and Health Survey 2018.

[5] De Onis M., Borghi E., Arimond M., Webb P., Croft T., Saha K., De-Regil L.M., Thuita F., Heidkamp R., Krasevec J. (2019). Prevalence thresholds for wasting, overweight and stunting in children under 5 years. Public Health Nutr..

[6] Markowitz D.L., Cosminsky S. (2005). Overweight and stunting in migrant Hispanic children in the USA. Econ. Hum. Biol..

[7] Fanzo J., Hawkes C., Udomkesmalee E., Afshin A., Allemandi L., Assery O., Baker P., Battersby J., Bhutta Z., Chen K. (2018). Global Nutrition Report: Shining a Light to Spur Action on Nutrition.

[8] Myatt M., Khara T., Schoenbuchner S., Pietzsch S., Dolan C., Lelijveld N. (2018). Children who are both wasted and stunted are also underweight and have a high risk of death: A descriptive epidemiology of multiple anthropometric deficits using data from 51 countries. Arch. Public Health.

[9] Ong K.K., Hardy R., Shah I., Kuh D. (2013). Childhood stunting and mortality between 36 and 64 years: The british 1946 birth cohort study. J. Clin. Endocrinol. Metab..

[10] Dewey K.G., Begum K. (2011). Long-term consequences of stunting in early life. Matern. Child Nutr..

[11] Mzumara B., Bwembya P., Halwiindi H., Mugode R., Banda J. (2018). Factors associated with stunting among children below five years of age in Zambia: Evidence from the 2014 Zambia demographic and health survey. BMC Nutr..

[12] Rakotomanana H., Gates G.E., Hildebrand D., Stoecker B.J. (2017). Determinants of stunting in children under 5 years in Madagascar. Matern. Child Nutr..

[13] Das S., Gulshan J. (2017). Different forms of malnutrition among under five children in Bangladesh: A cross sectional study on prevalence and determinants. BMC Nutr..

[14] Rajula H.S.R., Verlato G., Manchia M., Antonucci N., Fanos V. (2020). Comparison of conventional statistical methods with machine learning in medicine: Diagnosis, drug development, and treatment. Medicina.

[15] Iniesta R., Stahl D., McGuffin P. (2016). Machine learning, statistical learning and the future of biological research in psychiatry. Psychol. Med..

[16] Shahriar M., Iqubal M.S., Mitra S., Das A.K. A deep learning approach to predict malnutrition status of 0-59 month’s older children in Bangladesh. Proceedings of the 2019 IEEE International Conference on Industry 4.0, Artificial Intelligence, and Communications Technology (IAICT).

[17] Jin Z., Shang J., Zhu Q., Ling C., Xie W., Qiang B. (2020). RFRSF: Employee Turnover Prediction Based on Random Forests and Survival Analysis. Web Information Systems Engineering—WISE 2020.

[18] Markos Z., Doyore F., Yifiru M., Haidar J. (2014). Predicting Under Nutrition Status of Under-Five Children Using Data Mining Techniques: The Case of 2011 Ethiopian Demographic and Health Survey. J. Health Med. Inform..

[19] Talukder A., Ahammed B. (2020). Machine learning algorithms for predicting malnutrition among under-five children in Bangladesh. Nutrition.

[20] Bitew F.H., Sparks C.S., Nyarko S.H. (2021). Machine learning algorithms for predicting undernutrition among under-five children in Ethiopia. Public Health Nutr..

[21] Boyle E.H., King M., Sobek M. (2020). IPUMS-Demographic and Health Surveys: Version 8 [dataset].

[22] Pedregosa F., Varoquaux G., Gramfort A., Michel V., Thirion B., Grisel O., Blondel M., Prettenhofer P., Weiss R., Dubourg V. (2011). Scikit-learn: Machine Learning in Python. Scikit-Learn. Mach. Learn. Python.

[23] Lee W. (2019). Python^®^ Machine Learning.

[24] Cox D.R. (1958). The Regression Analysis of Binary Sequences. J. R. Stat. Soc. Ser. B.

[25] McCallum A., Nigam K. (1998). A comparison of event models for naive bayes text classification. AAAI-98 Workshop Learn. Text Categ..

[26] Zhang D. (2019). Bayesian Classification. Fundamentals of Image Data Mining.

[27] Pisner D.A., Schnyer D.M. (2019). Support Vector Machine.

[28] Lewes G.H., Awad M., Khanna R. (2015). Support Vector Machines for Classification. Efficient Learning Machines: Theories, Concepts, and Applications for Engineers and System Designers.

[29] Friedman J., Tibshirani R., Hastie T. (2000). Additive logistic regression: A statistical view of boosting (With discussion and a rejoinder by the authors). Ann. Stat..

[30] Nokeri T.C. (2022). Data Science Solutions with Python.

[31] Sheridan R.P., Wang W.M., Liaw A., Ma J., Gifford E.M. (2016). Extreme Gradient Boosting as a Method for Quantitative Structure-Activity Relationships. J. Chem. Inf. Model..

[32] Caruana R., Niculescu-Mizil A. Predicting good probabilities with supervised learning. Proceedings of the 22nd International Conference on Machine Learning.

[33] Python Software Foundation Python Language Reference. http://www.python.org.

[34] Mediani H.S. (2020). Predictors of Stunting Among Children Under Five Year of Age in Indonesia: A Scoping Review. Glob. J. Health Sci..

[35] Bwalya B.B., Lemba M., Mapoma C.C., Mutombo N. (2015). Factors Associated with Stunting among Children Aged 6–23 Months in Zambian: Evidence from the 2007 Zambia Demographic and Health Survey. Int. J. Adv. Nutr. Health Sci..

[36] Perumal N., Bassani D.G., Roth D.E. (2018). Use and misuse of stunting as a measure of child health. J. Nutr..

[37] Mhasawade V., Zhao Y., Chunara R. (2021). Machine learning and algorithmic fairness in public and population health. Nat. Mach. Intell..

[38] Deo R.C. (2015). Machine learning in medicine. Circulation.

